# Supplementary material for: Multi‐dataset identification of innovative feature genes and molecular mechanisms in keratoconus
Source: J Cell Mol Med. 2024 Sep 19;28(18):e70079. doi: 10.1111/jcmm.70079 (PMC11412914; doi:10.1111/jcmm.70079)
Supplement: Supplementary file 2 — Material S2. [file JCMM-28-e70079-s002.zip › 7.GSEA analysis/2.ATOH7/KEGG_PROXIMAL_TUBULE_BICARBONATE_RECLAMATION.html]

Details for gene set KEGG\_PROXIMAL\_TUBULE\_BICARBONATE\_RECLAMATION[GSEA]

|  || Dataset | ATOH7.group.cls#h\_versus\_l |
| Phenotype | group.cls#h\_versus\_l |
| Upregulated in class | h |
| GeneSet | KEGG\_PROXIMAL\_TUBULE\_BICARBONATE\_RECLAMATION |
| Enrichment Score (ES) | 0.44165036 |
| Normalized Enrichment Score (NES) | 1.3379835 |
| Nominal p-value | 0.064327486 |
| FDR q-value | 0.3341368 |
| FWER p-Value | 0.942 |
Table: GSEA Results Summary

  

Fig 1: Enrichment plot: KEGG\_PROXIMAL\_TUBULE\_BICARBONATE\_RECLAMATION      
 Profile of the Running ES Score & Positions of GeneSet Members on the Rank Ordered List

  

| SYMBOL | TITLE | RANK IN GENE LIST | RANK METRIC SCORE | RUNNING ES | CORE ENRICHMENT || 1 | SLC4A4 | na | 1125 | 0.429 | 0.1034 | Yes |
| 2 | CA2 | na | 1268 | 0.412 | 0.2296 | Yes |
| 3 | PCK2 | na | 2919 | 0.285 | 0.2721 | Yes |
| 4 | ATP1A2 | na | 3004 | 0.280 | 0.3584 | Yes |
| 5 | ATP1B2 | na | 4958 | 0.203 | 0.3663 | Yes |
| 6 | CA4 | na | 6349 | 0.161 | 0.3771 | Yes |
| 7 | PCK1 | na | 6929 | 0.145 | 0.4061 | Yes |
| 8 | GLS2 | na | 7210 | 0.138 | 0.4417 | Yes |
| 9 | ATP1B3 | na | 9532 | 0.090 | 0.4031 | No |
| 10 | ATP1A4 | na | 10390 | 0.075 | 0.4023 | No |
| 11 | ATP1A1 | na | 13481 | 0.036 | 0.3245 | No |
| 12 | GLUD2 | na | 13502 | 0.035 | 0.3351 | No |
| 13 | MDH1 | na | 15209 | 0.019 | 0.2920 | No |
| 14 | GLS | na | 16616 | 0.008 | 0.2540 | No |
| 15 | ATP1A3 | na | 17419 | 0.001 | 0.2312 | No |
| 16 | FXYD2 | na | 18133 | -0.005 | 0.2122 | No |
| 17 | AQP1 | na | 18196 | -0.006 | 0.2122 | No |
| 18 | SLC25A10 | na | 21838 | -0.041 | 0.1204 | No |
| 19 | GLUD1 | na | 22595 | -0.050 | 0.1142 | No |
| 20 | ATP1B1 | na | 23366 | -0.060 | 0.1110 | No |
| 21 | SLC38A3 | na | 27299 | -0.133 | 0.0399 | No |
| 22 | ATP1B4 | na | 30831 | -0.241 | 0.0143 | No |
| 23 | SLC9A3 | na | 32261 | -0.310 | 0.0711 | No |
Table: GSEA details [plain text format]

  

Fig 2: KEGG\_PROXIMAL\_TUBULE\_BICARBONATE\_RECLAMATION      
 Blue-Pink O' Gram in the Space of the Analyzed GeneSet

  

Fig 3: KEGG\_PROXIMAL\_TUBULE\_BICARBONATE\_RECLAMATION: Random ES distribution      
 Gene set null distribution of ES for **KEGG\_PROXIMAL\_TUBULE\_BICARBONATE\_RECLAMATION**
